# Estimating costs of bedside assessment by a judge in each case of mechanical restraint in Germany after new legislation

**DOI:** 10.3389/fpsyt.2023.1291130

**Published:** 2024-01-08

**Authors:** Sophie Hirsch, Erich Flammer, Tilman Steinert

**Affiliations:** Centers for Psychiatry Suedwuerttemberg, Ulm University, Ravensburg-Weissenau, Germany

**Keywords:** patient harm, economics, legislation and jurisprudence, emergencies, restraint

## Abstract

**Background:**

In 2018, the German Federal Constitutional Court decided that mechanical restraint is the most intrusive coercive measure and its use requires a judge’s decision after bedside assessment if lasting longer than 30 min. Subsequently, legal changes were realized. The objective of our study was to determine the number of saved coercive episodes and saved hours in seclusion or restraint in 2019 compared to the average of the previous years, 2015–2017, as well as costs per saved episode, hour, and case saved from any coercive measure.

**Methods:**

We used data from the Baden–Wuerttemberg case registry for coercive measures, covering all 32 psychiatric hospitals of the Federal State and 435,767 admissions in the study period. Time expenditure was calculated as 3.5 h with an average of 51.95 € per working hour on the side of the justice system and 1.5 h (45.94 €/h) on the side of the hospital per case.

**Results:**

The number of coercive episodes decreased by 10.0% from 28,181 (average 2015–2017) to 25,371 (2019). The number of hours in seclusion or restraint decreased by 17.9% from 321,956 (2015–2017) to 264,423 (2019). This resulted in the cost of 872.33 € per saved episode and 42.61 € per saved hour in seclusion or restraint.

**Conclusion:**

Given the correctness of our estimations, saving 1 h in coercion by less than 1 h of an expert’s work might be justified from an ethical and economic perspective.

## 1 Introduction

Coercive measures in psychiatric hospitals occur worldwide, based on laws to prevent danger against self or others in people with impaired mental capacity due to their mental disorder (1) despite ethical concerns. Reducing the use of coercion is deemed a priority in mental healthcare in terms of human rights and quality of treatment internationally (2), as well as in Germany (3). A wide range of evidence-based interventions is available to reduce the use of coercion (4). In Germany, comprehensive evidence- and consensus-based guidelines on the prevention of coercion in psychiatric facilities were published in 2018 (5).

### 1.1 Context

In 2018, the Federal Constitutional Court made a seminal decision after a 2-day hearing of experts that mechanical restraint is the most intrusive coercive measure, and therefore, its necessity has to be assessed by a judge at the patient’s bedside if lasting more than 30 min and that 1:1 supervision must be provided to users while they are in restraints. Subsequently, the laws of the Federal States were adopted accordingly in the course of the year 2018. In previous publications, we could show that the percentage of admitted patients subjected to any kind of freedom-restricting coercion decreased from 6.6% in 2017 to 5.8% in 2019 in the State of Baden-Wuerttemberg with 11 million inhabitants as a result of this legislative amendment (6, 7).

### 1.2 Key elements of the new legislation

Following the amendments to the law, some changes have occurred, especially in the judicial system. The on-call judge service is now only available from 6 a.m. to 9 p.m. but is now used much more frequently.

In 2019, 18.10 new judges’ positions and of 4.30 clerks positions were created after the court’s decision in the 11-million State of Baden-Wuerttemberg (8). In the hospitals, the adjustments were less evident, as funding for the positions depended on other conditional factors. But here, too, organizational adjustments had to be made to meet the requirements of the new laws. For the physicians, additional tasks result from the preparation of restraint requests. All clinical staff have to provide 1:1 care during existing restraints, which is very time-consuming, might be personally stressful, and places high quantitative and qualitative demands on personnel planning.

The legal changes had several effects, as shown by previous analyses (6): (i) A certain number of coercive measures probably did not occur under the new conditions of external review by a judge because clinicians dispensed with restraints. (ii) The duration of mechanical restraint decreased on average because the percentage of episodes lasting less than 30 min (and so, by law not requiring a judge’s review) increased from 1.8 to 10.5% because clinicians ended restraints earlier. (iii) To some extent, mechanical restraint was replaced by seclusion that served by and large the same purposes but did not need a review by a judge and, at least by law, no 1:1 supervision (6).

The objective of the study presented here was to determine the difference between coercive episodes and hours in any kind of freedom-restrictive measure (seclusion or restraint) in 2019 compared to the average of the previous years, 2015–2017, and the cost of these “saved” episodes and hours in seclusion or restraint by expenditures of the justice system and the clinical system.

## 2 Methods

### 2.1 Data recording

In 2015, a new mental health law was introduced in the German federal state of Baden-Wuerttemberg. It contained the unique feature of requiring all 32 psychiatric hospitals licensed to admit involuntary patients to collect data on seclusion, restraint, and forced medication in emergency situations or by judicial order. Raw data on each coercive measure in all hospitals are reported to the registry. For each coercive intervention, the dataset contains the kind of intervention as defined by a codebook, its legal basis, the duration, the patient’s gender, and the ICD-10 principal group. Hospitals must deliver data for the previous year before a deadline. The data are then checked for completeness and plausibility. In case of abnormalities, the clinics concerned are consulted, and if necessary and possible, the data are corrected. Further details of the registry have been reported elsewhere (9).

### 2.2 Outcomes

The main outcome was the amount of “saved coercive episodes,” which we determined (i) as the difference between the recorded episodes in seclusion or restraint in the average of the preceding years, 2015–2017, and in the year 2019 in absolute numbers. Deliberately, because of the effect of (iii), we did not use the numbers of restraint episodes but of coercive episodes of any kind (seclusion or restraint); otherwise, the effect would be overestimated. As a second outcome, we determined the difference of hours in seclusion or restraint in the average of the preceding years, 2015–2017, and in the year 2019. This outcome can be considered the most appropriate for the effectiveness calculation as it combines the effects of (i) and (ii) and is adjusted for the effects of (iii).

### 2.3 Cost estimates

All prices are calculated in Euro (€). Expenditures on the hospital side comprise the time for a psychiatrist to phrase the written request form to the court and to be present (in most cases at least) at the judge’s hearing. According to estimates of experts in the field in two different hospitals, the total amount of required time for physicians was estimated at 1.5 h on average. It would be complex to include clerks here as well. They are also not included in the re-funding of hospitals, so this seems to be a justifiable simplification. We calculated with average employer’s cost of 72,500 € (weighted average of senior and junior doctors on psychiatric or geriatric wards) multiplied by 1.2 for the employer’s contribution to social insurance per involved person/year and 1893.6 working h/year according to the federal average (250 working days, minus 31 days paid vacation, 5-days-working week, and 42 working hours per week), resulting in 45.94 € per working hour and, consequently, 68.91 € per reviewed restraint episode.

Expenditures on the side of the judicial system comprise the time for the journey from the court to the hospital and back, varying from a few minutes in some urban regions up to an hour in some rural regions, the time for talking with the patient and, possibly, staff, time for writing the decision, and time for office staff. PEBB§Y (10) is used for calculating personnel requirements for the German judicial authorities. The time allotted to complete these tasks varies from federal state to federal state. While, in Thuringia, 150 min of judge time are scheduled (11), in Hesse, it is between 104 and 219 min, depending on which judicial body is entrusted with the issue (12). There are no such benchmarks for Baden-Wuerttemberg so far, so we have estimated the figures after asking experts in the field (judges from two different courts, the Ministry of Justice in Baden-Wuerttemberg as well as consulting two PEBB§Y from two other states) by 3.5 h (210 min) on average. Subsequent time loss of judges by detrimental effects on their time schedule and other hearings canceled or delayed due to their unforeseen absence were not calculated. We calculated with average employer’s cost of 96,000 € (60,000 junior judges’ salaries to local courts in Baden-Wurttemberg plus 60% of pension liabilities) per involved person/year and 1848 working hours/year according to the federal average (250 working days, minus 30 days paid vacation, 5-days-working week, and 42 working hours per week), resulting in 51.95 € per working hour and, consequently, 181.82 € per reviewed restraint episode.

## 3 Results

**Figure 1:**
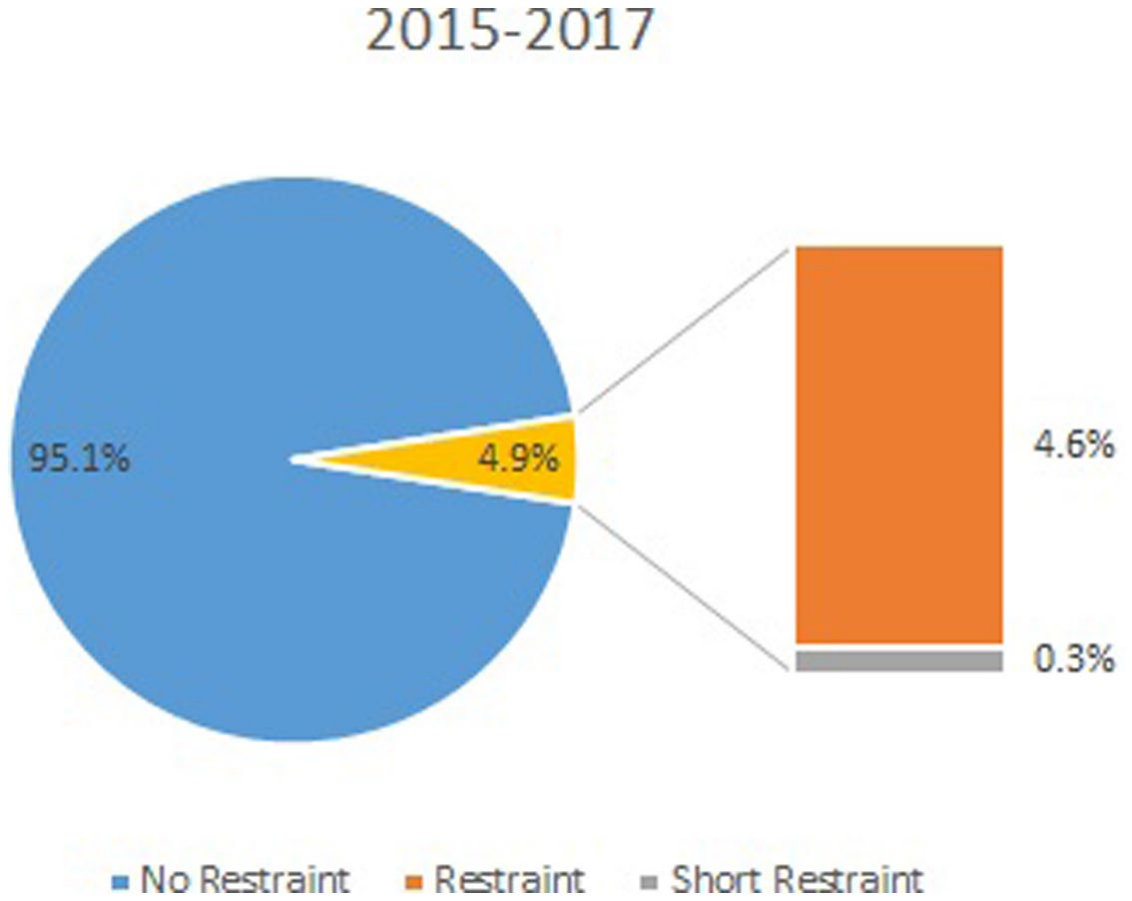
Percentage of cases with restraint for the years 2015–2017.

**Figure 2:**
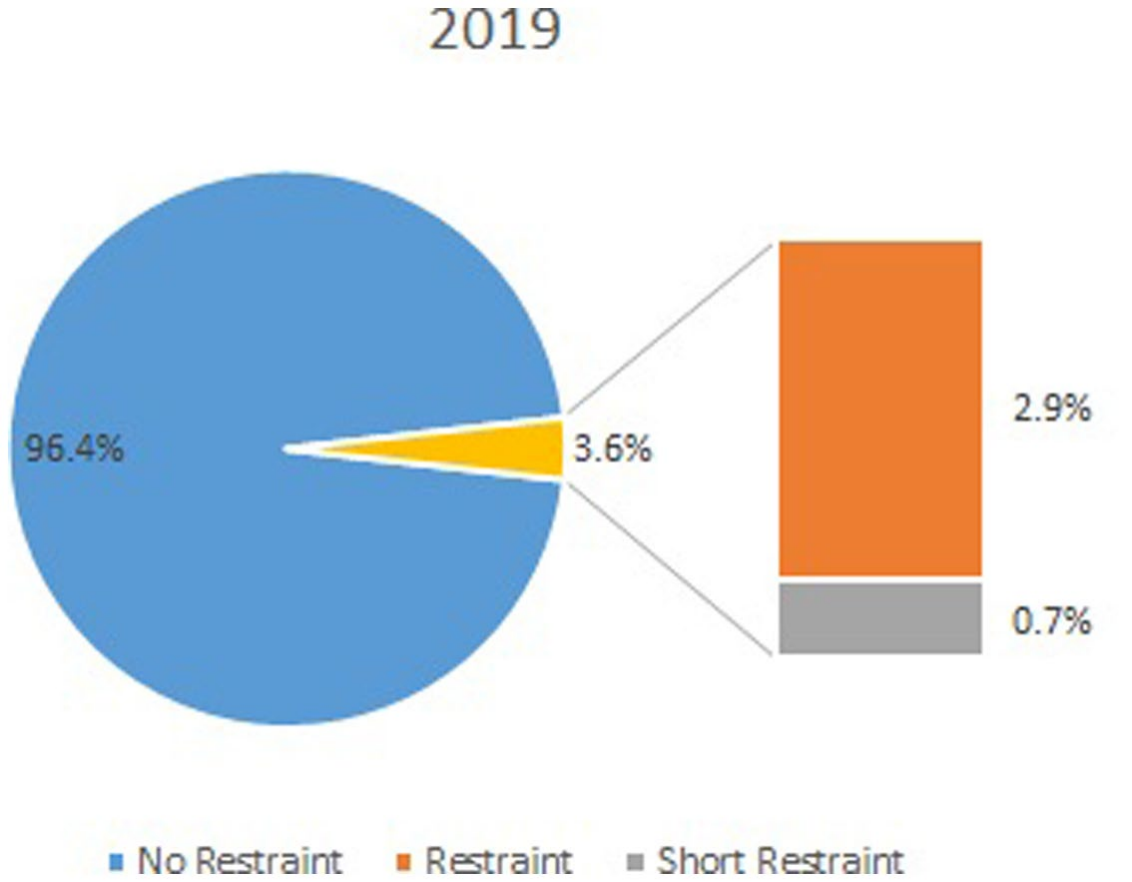
Percentage of cases with restraint for the year 2019.

The number and duration of episodes as well as the cost estimates are displayed in the table, comparing the average of the years, 2015–2017, and the year 2019. The lines in bold mark the defined outcomes (Table 1; Figures 1, 2).

**Table 1:** Comparisons of coercive interventions 2015–2017 and 2019.

	Average 2015–2017	2019	Saved
Cases	105,917	118,016	
**Cases affected by mechanical restraint or seclusion**	**7,135**	**6,814**	**321 (4.5%)**
Mechanical restraint episodes	17,554	10,923	
Cases affected by mechanical restraint	5,170	4,202	
Mechanical restraint episodes <30 min	332	1,147	
Cases affected by mechanical restraint episodes <30 min	327	769	
Mechanical restraint episodes >30 min reviewed by a judge	0	9,776	
Cases affected by mechanical restraint episodes >30 min reviewed by a judge	0	3,753	
Seclusion episodes	10,627	14,448	
**Episodes of seclusion or mechanical restraint**	**28,181**	**25,371**	**2,810 (10.0%)**
Hours in mechanical restraint	201,871	93,937	
Hours per mechanical restraint episode	11.5	8.6	
Hours in seclusion	120,085	170,486	
Hours per seclusion episode	11.3	11.8	
**Hours in seclusion or mechanical restraint**	**321,956**	**264,423**	**57,533 (17.9%)**
Hours per episode (any freedom-restrictive measure)	**11.4**	**10.4**	

The lines in bold mark the defined outcomes.

The expenditure in additional judges’ working hours for the 9.776 restraint episodes that needed review by a judge was 9,776 × 3.5 h = 34,216 h according to the estimations, resulting in the estimated cost of 1,777,521.20 € with 51,95 € per working hour. Hence, for each “saved” intervention (*N* = 2,810), the expenditure was 632.57 €; for each “saved” hour in coercion (57,533 h), 30.90 €. For each “saved” affected case (*N* = 321), the expenditure was 5,537.45 € (Table 2).

**Table 2:** Costs for reducing coercive measures in Euro, estimated by the salaries of average judges and physicians.

	Costs for judges	Costs for physicians	Total expenditures
Per decision	181.82	68.91	250.73
Per saved intervention	632.57	239.76	872.33
Per saved hour in coercion	30.90	11.71	42.61
Per saved affected case	5,537.45	2,098.64	7,636.09

The expenditure in additional physicians’ working hours for the 9,776 restraint episodes that needed review by a judge was 9,776 × 1.5 h = 14,664 h according to the estimations, resulting in the estimated cost of 673,726.24 € with 45.94 € per working hour. Hence, for each “saved” intervention (*N* = 2,810), the expenditure was 239.76 €; for each “saved” hour (57,533 h) in coercion, 11.71 €. For each “saved” affected case (*N* = 321), the expenditure was 2,098.64 €.

According to our estimates, 72.52% of the cost occurred on the side of the justice system, and 27.5% on the side of the health system.

## 4 Discussion

In this article, we estimated the cost of bringing about a decision to restrain 250.73 €. This includes 181.82€ for the judiciary and 68.91 € for the hospital. This reduction was not accompanied by an increase in forced medication, which increased insignificantly from 0.6 to 0.8% of treated patients during the observation period (6).

Our results show that the amount of hours in freedom-restrictive coercive interventions (seclusion or restraint) decreased by 17.9% in the year after the legal changes were realized. Due to the observational character of the data, this timely association is not necessary evidence of a causal association, but we are not aware of any other factor that could have changed the practice to such an extent. Therefore, we consider the publishing of the guidelines for the prevention of coercion (5), launched on the same day as the Federal Constitutional Court’s decision, as part of the intervention package whose single effects cannot be separated.

A reduced measure incurred costs of 872.33 €. To save a case from coercive measures, an average of 7,636.09 € was spent. To prevent 1 h in coercive measures, 42.61 € were spent.

Roughly spoken, less than 1 h of working time of a justice professional or health professional was required to save 1 h for a patient in a coercive measure. This can be considered a good investment not only for ethical but also for economic reasons, as 1:1 supervision by a professional is required by law in the case of mechanical restraint and, according to the guidelines, also in the case of seclusion. However, we have to be aware that there is no linear relationship. Probably, more external reviewing (it is difficult to imagine what would be more than a judge called to the patient’s bedside) would not yield a correspondingly stronger effect due to decreasing marginal utility or increasing marginal costs and maybe most critical: The resources on the hospital’s side that were consumed for the intervention—psychiatric specialists’ time capacities—are available only to a limited extent and cannot be recruited in the labor market currently. It means that the cost of the successful intervention was partly at the expense of fellow patients who were not subjected to coercive interventions and received less care, which imposes an ethical challenge as well as the question of whether and how opportunity costs can be taken into account (what could doctors and judges have been doing in that time, instead of reviewing and which other expenditures could have been done with the money).

Full cost data were provided by the Ministry of Justice Baden-Wuerttemberg for 2021 (respectively, 2022) for a decision. The Ministry of Justice reported 28,847 (27,578) decisions in cases of detention or measures involving restriction of freedom. According to this, 3,569 (3,386) cases were restrained under public law, which corresponds to a share of 12.4% (12.3%). With reported total costs of 10,629,510,16 € (10,888,505.27 €), it can therefore be assumed that the decision-making process for restraints costs approximately 1,315,101.11 € (1.336.880,08 €), which corresponds to 368.48 € (394.83 €) per restraint.

This corresponds to our results, at least in terms of the order of magnitude. As might be expected, the costs are higher. This is due to this full-cost calculation vs. only personnel costs in our approach. It is likely that the true costs lie between the two values, as even if costs for infrastructure, materials, etc., would have been taken into account (we treated them as sunk costs) in addition to personnel costs; on the other hand, restraints are relatively simple procedures and probably require somewhat fewer resources per case on average than other decisions, so that the equal allocation of full costs per decision probably overestimates the true costs.

## 5 Limitations

This is an observational study based on aggregated routine data, so a causal relationship between changes in the law and/or more judges and the reduction in coercive measures cannot be readily assumed. Especially the fact that the German clinical practice guidelines on the prevention of coercion were published on the same day as the ruling of the constitutional court might have led to contamination of the impact of the change in the law.

Even if a correlation is assumed, the economic conclusions are subject to a certain degree of uncertainty due to the lack of comparative data. We could not find any other reference values for upper and lower bounds on this topic. The EU-funded project PECUNIA, which among other objectives aims to make healthcare costs and costs from neighboring sectors (schools and justice) comparable across Europe, does not provide any standard values on coercive measures in psychiatry (13). This is probably due to the fact that the requirement of a judicial decision at the bedside is so far unique in Germany and does not occur in other European countries. PECUNIA takes “Lost freedom of offender” into account when measuring intersectoral costs and benefits as their outcomes. However, the project also states that this is one of the most unclear items (14) and seems to be difficult to operationalize.

## 6 Suggestions for further studies

As health economic approaches that only focus on the actual incurrence of monetary costs and returns (e.g., labor costs saved by less 1:1 care vs. additional labor costs due to more court cases, but also classical methods such as the human resources approach) are always subject to the criticism of underestimating the individual benefit for the individual patient, further studies examining people’s willingness to pay to avoid restraint would be interesting. However, further difficulties would arise here because the willingness to pay is not only correlated with the benefit but also with the ability to pay. Thus, a survey of the general population on this topic, e.g., after the presentation of pictures of restraints, would also be interesting. It would also be interesting to find out how patients experienced their hearing by a judge when they were already restrained. This could be done, for example, as part of structured debriefings following coercive measures.

## Data availability statement

The data analyzed in this study is subject to the following licenses/restrictions: the datasets generated for this study cannot be made publicly available. The data stored in the register is classified as confidential by the data protection officer of Baden-Wuerttemberg and is not publicly available due to data privacy. Requests to access these datasets should be directed to sophie.hirsch@zfp-zentrum.de.

## Ethics statement

The Ethics Committee of Ulm University waived the requirement for ethics approval as approval is not required for studies analyzing anonymized data, in accordance with national legislation and institutional requirements.

## Author contributions

SH: Formal analysis, Methodology, Validation, Writing – review & editing. EF: Data curation, Formal analysis, Methodology, Writing – review & editing. TS: Conceptualization, Supervision, Writing – original draft.
